# Understanding Racial Disparities in COVID-19–Related Complications: Protocol for a Mixed Methods Study

**DOI:** 10.2196/38914

**Published:** 2022-10-06

**Authors:** Jessica L Harding, Shivani A Patel, Teaniese Davis, Rachel E Patzer, Bennett McDonald, Doraina Walker-Williams, Ram Jagannathan, Larissa Teunis, Jennifer Gander

**Affiliations:** 1 Department of Surgery Emory University Atlanta, GA United States; 2 Department of Medicine Emory University School of Medicine Atlanta, GA United States; 3 Department of Epidemiology Rollins School of Public Health Emory University Atlanta, GA United States; 4 Hubert Department of Global Health Rollins School of Public Health Emory University Atlanta, GA United States; 5 Center for Research and Evaluation Kaiser Permanente Atlanta, GA United States; 6 Division of Hospital Medicine Emory University School of Medicine Atlanta, GA United States

**Keywords:** COVID-19, social determinants of health, race, mixed methods, equity, disparity, health, pandemic, disease severity, mortality, racial, ethnicity, complications

## Abstract

**Background:**

In the United States, the COVID-19 pandemic has magnified the disproportionate and long-standing health disparities experienced by Black communities. Although it is acknowledged that social determinants of health (SDOH) rather than biological factors likely contribute to this disparity, few studies using rigorous analytic approaches in large, information-rich community-based data sets are dedicated to understanding the underlying drivers of these racial disparities.

**Objective:**

The overall aim of our study is to elucidate the mechanisms by which racial disparities in severe COVID-19 outcomes arise, using both quantitative and qualitative methods.

**Methods:**

In this protocol, we outline a convergent parallel mixed methods approach to identifying, quantifying, and contextualizing factors that contribute to the dramatic disparity in COVID-19 severity (ie, hospitalization, mortality) in Black versus white COVID-19 patients within the integrated health care system of Kaiser Permanente Georgia (KPGA). Toward this end, we will generate two quantitative cohorts of KPGA members with a confirmed COVID-19 diagnosis between January 1, 2020, and September 30, 2021: (1) an electronic medical record (EMR) cohort including routinely captured data on diagnoses, medications, and laboratory values, and a subset of patients hospitalized at Emory Healthcare to capture additional in-hospital data; and (2) a survey cohort, where participants will answer a range of questions related to demographics (eg, race, education), usual health behaviors (eg, physical activity, smoking), impact of COVID-19 (eg, job loss, caregiving responsibilities), and medical mistrust. Key outcomes of interest for these two cohorts include hospitalization, mortality, intensive care unit admission, hospital readmission, and long COVID-19. Finally, we will conduct qualitative semistructured interviews to capture perceptions of and experiences of being hospitalized with COVID-19 as well as related interactions with KPGA health care providers. We will analyze and interpret the quantitative and qualitative data separately, and then integrate the qualitative and quantitative findings using a triangulation design approach.

**Results:**

This study has been funded by a Woodruff Health Sciences grant from December 2020 to December 2022. As of August 31, 2022, 31,500 KPGA members diagnosed with COVID-19 have been included in the EMR cohort, including 3028 who were hospitalized at Emory Healthcare, and 482 KPGA members completed the survey. In addition, 20 KPGA members (10 Black and 10 white) have been interviewed about their experiences navigating care with COVID-19. Quantitative and qualitative data cleaning and coding have been completed. Data analysis is underway with results anticipated to be published in December 2022.

**Conclusions:**

Results from this mixed methods pilot study in a diverse integrated care setting in the southeastern United States will provide insights into the mechanisms underpinning racial disparities in COVID-19 complications. The quantitative and qualitative data will provide important context to generate hypotheses around the mechanisms for racial disparities in COVID-19, and may help to inform the development of multilevel strategies to reduce the burden of racial disparities in COVID-19 and its ongoing sequelae. Incorporating contextual information, elucidated from qualitative interviews, will increase the efficacy, adoption, and sustainability of such strategies.

**International Registered Report Identifier (IRRID):**

RR1-10.2196/38914

## Introduction

### Background

In the United States, the COVID-19 pandemic has magnified the disproportionate and long-standing health disparities experienced by Black communities. Substantive data now demonstrate that Black Americans experience infection, hospitalization, and death from COVID-19 at disproportionality high rates [1-5]. For example, in the state of Georgia, Black Americans represent 31% of the population, yet they account for approximately 40% of total COVID-19 deaths [6]. Now, as we approach our third year of the pandemic, an abundance of extant literature points to the heavily racialized effects of COVID-19, yet there has been scarce discourse and few interventions addressing the disproportionate toll among Black populations due to a lack of actionable evidence needed to inform such responses. Unpacking the role of structural racism (through the multilevel processes that interact with one another to generate and reinforce disparities faced by racialized communities) on the risk of COVID-19 complications, including severe COVID-19 infections requiring hospitalization and “long COVID-19,” remains crucial to inform pandemic responses among Black communities.

Social determinants of health (SDOH), rather than biological differences, are hypothesized to impose a greater risk for both infection and severe disease from COVID-19 (ie, hospitalization) among Black communities [7]. These include a myriad of factors operating at the level of the individual (eg, chronic disease burden), interpersonal (eg, patient-provider relationship), community (eg, health care availability), and social and economic structure (eg, poverty rate, racial segregation). Although it is acknowledged that these factors likely contribute, few studies using rigorous analytic approaches in large, information-rich community-based data sets are dedicated to understanding the underlying drivers of these racial disparities. 

### Objective

**Figure 1:**
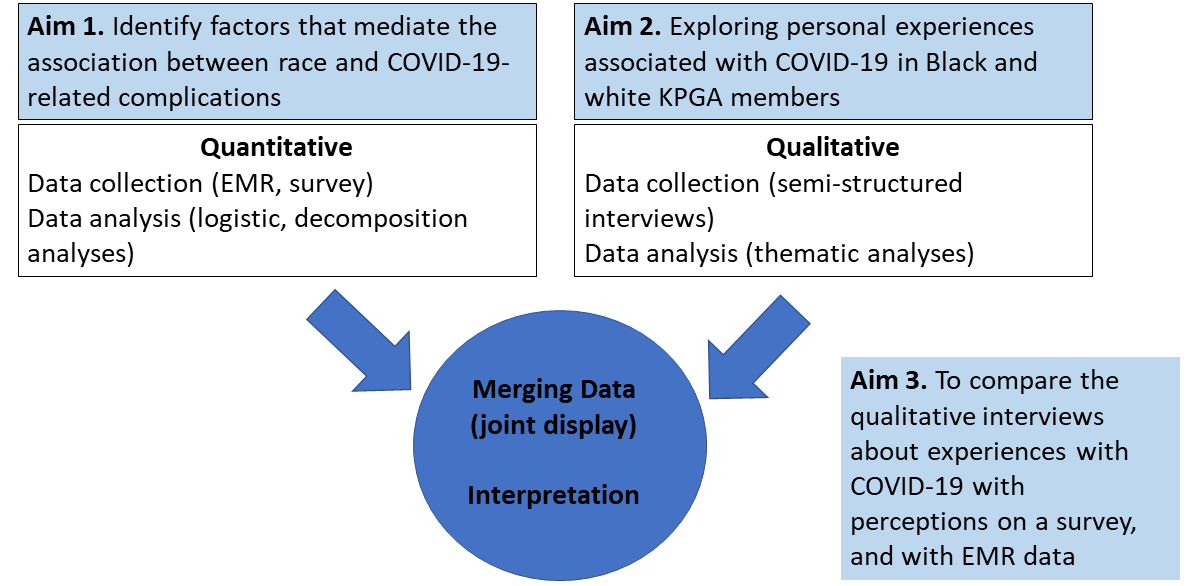
Convergent parallel mixed methods design to understand racial disparities in COVID-19–related complications. EMR: electronic medical record; KPGA: Kaiser Permanente Georgia.

In this protocol, we outline a mixed methods approach to identifying, quantifying, and contextualizing the specific medical and SDOH factors that contribute to the dramatic disparity in COVID-19 complications in Black versus white COVID-19 patients within an integrated health care system. The specific aims of this planned research are to: (1) quantitatively examine the individual, community, and structural factors contributing to (ie, mediating) disparities in COVID-19 complications in Black versus white COVID-19 patients using electronic medical record (EMR) data and primary survey data; (2) conduct semistructured qualitative interviews among Black and white patients hospitalized with COVID-19 to explore personal experiences with COVID-19, and contextualize factors that facilitate and impede health-seeking behaviors at the interpersonal, family, community, and health care levels; and (3) compare and contrast the qualitative interviews about personal experiences with COVID-19 with perceptions on the quantitative survey and routinely collected EMR data (Figure 1). This mixed methods approach will provide a robust understanding of the multifactorial challenges faced by adults diagnosed with COVID-19, and compare these challenges between Black and white patients to inform future interventions and policies that may reduce barriers and improve equity.

## Methods

### Conceptual Framework

Our approach is informed by the National Institute of Minority Health and Health Disparities (NIMHD) Research Framework (Table 1) [8]. This framework considers the complex interplay among individual, interpersonal, community, and structural factors that influence health and health outcomes. In this study, the NIMHD Framework informed our quantitative EMR cohort and survey development, as well as the qualitative interview guide.

**Table 1:** The National Institute on Minority Health and Health Disparities Research Framework [8].

Levels of influence	Domains of influence				
	Biological	Behavioral	Physical/built environment	Sociocultural environment	Health care system
Structural	Population exposure	Policies and laws (eg, social distancing)	Societal structure	Societal norms; society; structural discrimination; media	Quality of care; health care policies
Community	Community exposure	Community functioning	Community environment; community resources	Community norms; local structural discrimination	Availability of health services; safety net services
Interpersonal	Family microbiome; caregiver-child interaction	Family functioning; school/work functioning	Household environment; school/work environment	Social networks; family/peer norms; interpersonal discrimination	Patient-provider relationship; medical decision-making
Individual	Preexisting conditions	Health behaviors (including social distancing); coping strategies	Personal environment	Sociodemographic; cultural identity; response to discrimination	Insurance coverage; health literacy; trust in health care system

### Community Advisory Board

Evidence suggests that a community-engaged approach leads to the development of more efficacious and readily adoptable interventions [9], the long-term objective of our work. For this study, we have formed a community advisory board (CAB; N=5) comprised of patients, caregivers, and researchers. CAB members were recruited through established community engagement networks, academic institutions, local community organizations, and health care systems. The composition of the CAB is 70% women and 100% nonwhite. To date, the CAB has helped inform the development of the qualitative interviews. It is anticipated that results arising from the study will be disseminated to the CAB, which will be essential to contextualizing results and informing the development future multilevel intervention studies to reduce COVID-19–related racial disparities**.**

### Study Population and Data Sources

#### Kaiser Permanente Georgia

Kaiser Permanente Georgia (KPGA) is a large health insurance database of more than 260,000 current adult members (>40% Black) across 2230 US Census tracts in the metropolitan Atlanta area as well as North Georgia. To be enrolled in the database, participants must have insurance with KPGA. The large proportion of Black members (in the general Georgia population, the proportion of people identifying as Black is 32.6%) and variability in SDOH indices (household income, social vulnerability index) will allow us to investigate racial disparities and effect modification by individual circumstances, health care site, and neighborhood. KPGA has an extensive EMR data repository, including information related to patient demographics (with some individual measures of SDOH such as insurance status), diagnoses, procedures, claims, lab values, and prescribed medications. In addition, community-level SDOH variables were drawn from an extensive database of characteristics at the county, census-tract, and zip code levels to characterize social vulnerability factors at the community and system levels. Data on community- and system-level factors were obtained from publicly available sources (eg, American Community Survey), which were geocoded and linked to patient EMR data using information of the patient address.

#### EMR Cohort (Quantitative)

To develop the EMR cohort, all adult (aged ≥18 years) members enrolled in KPGA as of January 1, 2020, with a minimum of 1-month continuous enrollment and with a confirmed diagnosis of COVID-19 were included (N=31,500). COVID-19 was defined by a positive COVID-19 polymerase chain reaction test or an International Classification of Diseases-10th revision (ICD-10) diagnosis code (U07.1, B97.29, B34.2, B97.21, or J12.81). To ascertain granular information on in-hospital outcomes (eg, intensive care unit [ICU] admission), KPGA EMR data were linked to Emory Healthcare for the subset of KPGA members hospitalized with COVID-19 at Emory Healthcare (n=3028). KPGA does not offer inpatient services and Emory Healthcare represents >50% of all hospitalizations among KPGA members in metropolitan Atlanta. Linkage of KPGA to Emory Healthcare data was done using an algorithm of date of birth, first name, last name, and sex, with a linkage rate greater than 90%.

#### COVID-19 Survey Cohort (Quantitative)

For the COVID-19 survey, adult (aged ≥18 years) KPGA members with a confirmed COVID-19 diagnosis; a valid email address; and current KPGA enrollment with a minimum of 1-month continuous enrollment as of June 1, 2021, were invited to participate via email. The cohort eligible for the survey was populated on June 1, 2021, and research staff began emailing eligible adults a recruitment email with an embedded survey link. Emails were sent in batches of 500 between July 1, 2021, and August 15, 2021. In total, 482 people completed the survey with a response rate of 3%, similar to other Kaiser Permanente email-administered surveys. All participants provided informed consent.

#### Interview Cohort (Qualitative)

For semistructured interviews, Black and white adult (aged ≥18 years) KPGA members with a confirmed COVID-19 diagnosis and hospitalized with COVID-19 with a discharge date between March 2020 and March 2021 were eligible to be recruited. KPGA members were recruited via the KPGA patient portal (Health Connect), email, phone, and mail. Upon initial contact, we additionally screened individuals to ensure we only recruited those who self-identify as Black or white and ensured an equal distribution of participants by race (ie, 10 Black and 10 white participants). Using this recruitment method, and anticipating a 10%-20% response rate [10], we invited approximately 200 KPGA members to achieve our sample size of 20. Based on guidance, completing 20 interviews among a racially balanced cohort will be adequate for ensuring an appropriate saturation of themes [11].

Table 2 describes the four distinct populations in this study, and respective measurements and study outcomes.

**Table 2:** Study populations, measurements, and outcomes of interest.

Study population			Study population description		Participants, n		Measurement(s)		Outcome(s)
**Quantitative**									
	EMR^a^ cohort	All adult KPGA^b^ members diagnosed with COVID-19 between January 1, 2020, and June 1, 2021		31,500		EMR data, including demographics, neighborhood-level SDOH^c^, comorbidities, medications, and lab values		Hospitalization within 30 days of COVID-19 diagnosis; readmission (30, 60, and 90 days); mortality; long COVID	
	Survey cohort	We invited those in the EMR cohort with a valid email address (n=~17,500) to complete a COVID-19 survey		482		Survey questions related to demographics (eg, race, education), usual health behaviors (eg, physical activity, smoking), impact of COVID-19 (eg, job loss, caregiving responsibilities), and medical mistrust. Survey data were supplemented with EMR data		Hospitalization within 30 days of COVID-19 diagnosis; readmission (30, 60, and 90 days); mortality; long COVID	
	KPGA-Emory cohort	All adult KPGA members hospitalized at Emory Healthcare with COVID-19 between January 1, 2020, and June 1, 2021		3028		KPGA EMR data, supplemented with data on in-hospital medications and lab values from Emory Healthcare		In-hospital outcomes: mechanical ventilation, COVID-19 treatment, ICU^d^ admission, ECMO^e^ use	
Qualitative: interview cohort			We invited those in the EMR cohort with a valid email address (n=~17,500) to participate in a 60-minute one-on-one interview		10		Semistructured interviews		Themes

^a^EMR: electronic medical record.

^b^KPGA: Kaiser Permanente Georgia.

^c^SDOH: social determinants of health.

^d^ICU: intensive care unit.

^e^ECMO: extracorporeal membrane oxygenation.

### Quantitative Methods and Analysis: EMR Cohort

#### Primary Exposure: Race

Race is a social construct describing groups that have associated racial meanings that affect their economic, political, and social lives [7,12]. Racial inequalities are influenced by class differences and SDOH [12,13]. In this study, the primary independent variable will be race, determined from KPGA patient self-report data, and will focus on Black and white adults. Based on guidance by Ioannidis et al [14] and Lin and Kesley [15], the use of race in the current context is appropriate, as other SDOH factors often fail to associate (with sufficient precision) when race is used as the placeholder, and the development of our models will carefully consider other explanatory biological and sociologic variables that may explain race-based signals. Further, due to persistent structural inequities that exist across multiple levels, studying the magnitude of disparities between Black and white individuals in EMR data is often difficult because of missing race/ethnicity data. Therefore, to address missing data on self-identified race (~24% among adults in KPGA), we will apply a Bayesian method integrating surname and geocoded information to impute self-reported race [16]. This approach has previously shown high correlation (76%) with self-reported race with other Kaiser Permanente databases [16]. Analyses will be performed with and without imputed race. Quantitative findings of factors contributing to racial disparities will be merged with the perceptions and experiences from semistructured interviews using a triangulation design. Of note, the current study protocol is restricted to examine differences between Black and white individuals and does not include other racial or ethnic groups, or those identifying as multiracial. This is because the reasons for racial and ethnic disparities in health outcomes across groups are complex and must be carefully considered against each group’s historical, social, and economic circumstances. Here, we focus on Black versus white disparities to better ensure that the research provides specific and actionable insight for this important subgroup. Future work will incorporate other racial and ethnic groups.

#### Covariates

A list of the multilevel variables that will be considered as confounders and/or mediators based on our conceptual model, along with their respective data sources, is detailed in Table 3. We will consider these variables in the context of individual-, community-, and system-level factors, but acknowledge that these are not always mutually exclusive and that many risk factors have upstream causes for which solutions should also be upstream.

**Table 3:** Quantitative study variables and data sources.

Independent variables		Data Source
**Individual-level factors**		
	Demographics (eg, race, age, sex, ethnicity)	KPGA^a^ EMR^b^
	Insurance coverage	KPGA EMR
	Primary language spoken at home	KPGA EMR
	COVID-19 diagnosis date	KPGA EMR
	Pre-existing conditions	KPGA EMR
	Medical treatment	KPGA EMR
	Vital signs and lab data	KPGA EMR
	In-hospital lab values	Emory Healthcare
	In-hospital medications	Emory Healthcare
	Marital status	COVID-19 Survey
	SDOH^c^ (eg, education, household income)	COVID-19 Survey
	Locus of control	COVID-19 Survey
	Health behaviors (eg, exercise, smoking, drinking) pre- and post-COVID-19	COVID-19 Survey
	COVID-19 symptoms	COVID-19 Survey
	Health care–seeking behavior	COVID-19 Survey
	Impacts of COVID-19 pandemic (eg, job loss)	COVID-19 Survey
	Vaccine hesitancy	COVID-19 Survey
	Medical mistrust	COVID-19 Survey
**Community- and structural-level factors**		
	Neighborhood deprivation index	American Community Survey
	Median household income	American Community Survey
	Social vulnerability index	American Community Survey
**Outcome variables**		
	30-day hospitalization	KPGA EMR
	Readmission (30-day, 60-day, 90-day)	KPGA EMR
	ICU^d^ admission	Emory Healthcare
	Mechanical ventilator use	Emory Healthcare
	COVID-19 treatment	Emory Healthcare
	ECMO^e^ use	Emory Healthcare
	Long COVID complications: cardiovascular (CAD^f^, HF^g^, MI^h^, stroke, PVD^i^); respiratory (fibrotic lung disease, bronchiectasis, pulmonary vascular disease; mental health (depression, anxiety, substance abuse)	KPGA EMR
	Mortality	KPGA EMR

^a^KPGA: Kaiser Permanente Georgia.

^b^EMR: electronic medical record.

^c^SDOH: social determinants of health.

^d^ICU: intensive care unit.

^e^ECMO: extracorporeal membrane oxygenation.

^f^CAD: coronary artery disease.

^g^HF: heart failure.

^h^MI: myocardial infarction.

^i^PVD: peripheral vascular disease.

#### Outcomes

##### COVID-19–Related Hospitalizations

Hospitalizations will be considered to be COVID-19–related if they occurred within 30 days of the COVID-19 diagnosis date and include an ICD-10 code for COVID-19.

Hospital Readmissions

Hospital readmissions will be defined as readmissions at 30, 60, and 90 days following the first hospital discharge date.

##### In-Hospital Outcomes

ICU admission, COVID-19 treatment, and ventilator status will be defined based on KPGA and Emory Healthcare data

##### Long COVID

Long COVID will be defined through multiple outcome domains: cardiovascular (coronary artery disease, heart failure, myocardial infarction, stroke, peripheral vascular disease); metabolic (diabetes); kidney (acute kidney injury); respiratory (fibrotic lung disease, bronchiectasis, pulmonary vascular disease); mental health (depression, anxiety, substance abuse). Long COVID outcomes will be defined using ICD-10 codes as appropriate. To minimize misclassification of acute COVID-19 complications, as well as previously undiagnosed conditions, long COVID will be defined as symptoms >30 days following the initial COVID-19 infection date.

##### Mortality

Vital status is updated on a quarterly basis by a dedicated team at KPGA. We will consider COVID-19–specific deaths and all-cause deaths in this group (Tables 2-3).

#### Statistical Analysis

##### Overview

In this open cohort study, we will follow individuals in our cohort from the date of first COVID-19 infection, through to each outcome of interest (ie, hospitalization, postacute sequelae of COVID-19, death, or end of enrollment). All primary analyses will consider time to first event (ie, first COVID-19–related event). In sensitivity analyses, we will consider multiple events (ie, >1 event). In addition, given the various waves of COVID-19 (ie, emergence of the Delta and Omicron variants), all analyses will be stratified by calendar period.

##### Summary Statistics

The study population characteristics will be described with summary statistics as appropriate for the EMR cohort. The *χ*^2^, *t*, and Wilcoxon rank-sum tests will be used to test for differences in baseline characteristics by race as appropriate. We will fit multivariable Poisson regression models, negative binomial regression, and generalized Poisson regression to estimate the excess risk of COVID-19 outcomes in Black versus white adults, and determine the multilevel factors associated with this excess risk using a stepwise approach [17]. All models will consider variability across calendar time. Given the known sex disparity in COVID-19 (ie, men have higher risk of severe COVID-19 compared with women) [18], we will additionally stratify all results by sex. Findings will also be stratified by age and vaccination status to examine the effect modification on their association with severe COVID-19 outcomes. Study variables obtained from EMR data, excluding race, are expected to be available for >95% of participants based on prior analyses. Therefore, our primary approach will be a complete case analysis. However, we will perform sensitivity analyses using hot-deck imputation, replacing missing values with imputed values as estimated from respondents with matching covariates [19].

##### Decomposition Analysis

Following a social-ecological approach, we will apply the Oaxaca-Blinder decomposition technique to quantify the contribution of individual, community, and structural exposures to racial disparities in COVID-19 outcomes. This regression-based counterfactual method was originally developed in economics with recent applications in epidemiology [20,21]. We will use this method to partition the disparity in outcomes between Black and white KPGA members into the portion that is explained by differences in the levels of exposures across race, differences in the associations of the exposures across race, and the portion that is unexplained by exposures included in the model (ie, other unmeasured factors such as racism). The output from the decomposition analysis will provide insight on the expected residual disparity in outcome if Black and white adults experienced the same level of exposures (eg, equal health care access), sample exposure effects (eg, equal effects on outcomes once health care is accessed), and the interaction between level and effects of exposures. This technique will enable us to quantify the confounder-adjusted potential impact of targeting specific exposures and exposure combinations (which may be differentially distributed by race but also have differential effects on outcomes for each race) on the Black-white disparity in study outcomes. This quantification can be used to prioritize future intervention efforts. Finally, effect modification by area-level characteristics will be evaluated through stratified decomposition analysis among adults residing in counties with high and low vulnerability scores following established percentile-based indices (high: >75th percentile). All analyses will be performed using Stata version 16.1 (StataCorp).

##### Sample Size and Power Calculation

The EMR cohort is expected to follow 31,500 adults (~47.2% Black). For the rarest outcome, COVID-19 mortality (159 per 100,000 white adults) [22], we expect to be able to detect relative risks (between Black and white adults) of 1.4 with 0.9 power at the 5% significance level. Based on previous applied studies using decomposition analysis (sample size range 24 to 22,666,142), our study will have a modest sample size to conduct decomposition analysis, and based on a range of uncertainty estimates, we anticipate having 80% assurance for 80% power or higher [23].

### Quantitative Methods and Analysis: COVID-19 Survey Cohort

#### Survey Development

We collected additional individual-level patient information on COVID-19–positive patients via an electronic survey to explore specific factors, including SDOH, that may be associated with COVID-19 complications not captured in EMR data. Variables included in the survey (see Multimedia Appendix 1 and Table 3) were based on a priori knowledge as well as emerging questions specific to COVID-19, and obtained from a variety of sources, including the National Institute of Health’s Office of Behavioral and Social Sciences Research resource list of COVID-19–relevant domains for clinical or population research [24]. The survey, administered through Emory University’s RedCap system, was pilot-tested among a sample (N=15) of non-COVID-19 non-KPGA members, and estimated to take, on average, 8 (range 5-10) minutes to complete.

#### Race

Race, as described above for the EMR cohort, will be collected via self-report on the survey. We will define individuals as non-Hispanic Black and non-Hispanic white. Within the design of the survey, all individuals must answer questions on race before progressing in the survey. Therefore, we do not have any missing data on race for the COVID-19 survey.

#### Outcome

KPGA members who completed the COVID-19 survey were linked to the KPGA EMR using name, date of birth, and medical record number with an almost 100% match rate. This means that all COVID-19 survey participants will also have EMR data on comorbidities, lab values, and medications as per the EMR cohort. The primary outcome for COVID-19 survey participants will be 30-day hospitalization as ascertained by the KPGA EMR (Table 3).

#### Statistical Analysis

##### Overview

The analytic approach for the COVID-19 survey cohort will be similar to that described for the EMR cohort under the Summary Statistics subsection above. We do not have sufficient power to perform a decomposition analysis on this sample.

##### Sample Size and Power Calculation

Using a Poisson regression for our primary outcome of COVID-19–related hospitalization within 30 days of infection among 482 survey participants, 38.6% of whom identify as Black, we estimate having 93% power at a .05 significance level to detect a minimum relative risk of 1.4. This sample size estimate is adjusted for covariates of age, gender, neighborhood vulnerability index, and median income.

### Qualitative Methods and Analysis

#### Overview

Examining racial disparities in COVID-19 using large EMR systems such as KPGA will provide quantitative data to explore the contribution of several multilevel factors to known racial disparities. However, this approach in isolation may overlook the complex interaction of contextual factors, cultural and personal values, social resources, and individual motivations that influence a person’s ability to seek health care and navigate the health care system. Therefore, this mixed methods project concurrently conducted in-depth semistructured interviews, guided by the theoretical framework outlined in Table 1 and with feedback from the CAB, to capture perceptions of and experiences of being hospitalized with COVID-19 as well as related interactions with KPGA health care providers. Qualitative methods such as this are well-suited to produce rich, contextual information from individuals deemed knowledgeable about specific issues [25]. Furthermore, capturing the patient experience and integrating this information into the design and development of future interventions, as our long-term objective, is known to increase the efficacy, adoption, and sustainability of such interventions [26].

#### Data Collection

Semistructured interviews were conducted among a cohort of 10 Black and 10 white participants diagnosed with COVID-19. According to the principles of qualitative research, we believe that a sample size of 20 will be sufficient to reach saturation for thematic analyses [27]. All interviews were conducted via telephone and audio-recorded. Each interview lasted ~60 minutes and was conducted by trained social behavioral scientists at KPGA with extensive experience in qualitative interviewing. The interview guide focused on factors related to health disparities and the multilevel factors associated with racial disparities in health and health care. This includes social environment (neighborhood-level access to quality care), medical mistrust, patient-provider interaction, and changes in employment or housing circumstances (Multimedia Appendix 2). We included a process for referring participants to counseling through KPGA’s Behavioral Health Department for any patients who report challenges during the interview. The interview guide and procedures were pilot-tested with a subset (n=2-3) of the study population prior to enrolling study participants. Participants were offered a nominal financial incentive (US $20 Amazon gift card) for participating in the interview.

#### Data Analysis

Semistructured interviews were audio-recorded and transcribed verbatim by two trained research assistants (one identifying as Black and the other as white) and overseen by a trained social behavioral scientist (identifying as Black). A random sample of transcripts were checked against the audio recordings for accuracy. We then developed a codebook using open coding to identify themes that emerged, followed by axial coding to categorize the themes that emerged to code the interview transcripts [26]. Two coders independently coded each interview transcript and any discordance between the primary coders was discussed with the group until a resolution was reached. Intercoder agreement will be assessed using κ values. We used NVivo 12.0 software to code data and organize results. Thematic analysis will be used to describe themes within the study domains and constructs. We will use modeling techniques to visualize relationships between themes that emerge among each group of participants.

### Mixed Methods Integration

This study will follow the checklist for mixed methods research proposed by Fetters and Molina-Azorin [28]. We will analyze and interpret quantitative and qualitative data separately, and then integrate the qualitative and quantitative findings using a triangulation design approach to directly compare and contrast quantitative statistical results with qualitative findings, and to validate quantitative findings with qualitative data. We will present quantitative data and qualitative data separately, and together in a joint display table (Figure 1).

### Ethics Approval and Dissemination

The KPGA Institutional Review Board (IRB; #00000406) and Emory University IRB (#MOD004-STUDY00001631) reviewed and approved this study. Online informed consent was obtained from all participants in the survey cohort. Verbal informed consent was obtained from all participants involved in qualitative interviews.

The Emory and KPGA IRBs waived the requirement of written Privacy Rule Authorization for use of protected health information for recruitment purposes, for the secondary data analysis portion of the study, and waived the requirement of written Privacy Rule authorization and the requirement to obtain a signed consent form for the survey and interview portions of the study.

Study findings will be disseminated with key stakeholders, including CAB members, KPGA, and Emory Healthcare, and will be presented at academic conferences and published in peer-reviewed journals.

### Data and Material Availability

The data that support the findings of this study are available from KPGA, but restrictions apply to the availability of these data, which were used under license for this study and so are not publicly available. However, data are available from the authors upon reasonable request and with permission of KPGA.

## Results

This study has been funded by a Woodruff Health Sciences grant from December 2020 to December 2022. As of August 31, 2022, 31,500 KPGA members diagnosed with COVID-19 between January 1, 2020, and September 30, 2021, have been included in the EMR cohort, including 3028 who were hospitalized at Emory Healthcare, and 482 KPGA members completed the survey. In addition, 20 KPGA members (10 Black and 10 white) have been interviewed about their experiences navigating care with COVID-19. Quantitative and qualitative data cleaning and coding have been completed. Data analysis is underway with results anticipated to be published in December 2022.

Table 4 describes the basic demographics of our three distinct study populations. In brief, the EMR cohort was more likely to be Black, female, and younger as compared to the general KPGA population. The survey cohort was less likely to be Black and male, and more likely to be older as compared to the general KPGA population. Finally, the interview cohort was more likely to be Black and male relative to the general KPGA population.

**Table 4:** Demographic characteristics of the three unique study populations diagnosed with COVID-19 included in this mixed methods study as compared to the general KPGA population

Characteristics	EMR^a^ cohort	Survey cohort	Interview cohort	KPGA^b^ population
Participants recruited, n	31,500	482	20	264,681
Black, n (%)	14,868 (47.2)	186 (38.6)	10 (50.0)	110,107 (41.6)
Men, n (%)	13,261 (42.1)	157 (32.5)	12 (60.0)	120,430 (45.5)
Aged>60 years, n (%)	5260 (16.7)	192 (39.8)	0 (0)	63,259 (23.9)

^a^EMR: electronic medical record.

^b^KPGA: Kaiser Permanente Georgia.

## Discussion

### Principal Findings

Results from this mixed methods pilot study in a diverse integrated care setting in the southeastern United States will provide insights into the mechanisms underpinning racial disparities in COVID-19 complications. We hypothesize that Black KPGA members will have an increased risk for COVID-19–related complications such as hospitalization, ICU admission, and ventilator use relative to white KPGA members. We also anticipate that a higher proportion of comorbidities among Black KPGA members will explain some, but not all, of the observed disparities, and that SDOH, including racism, will also contribute significantly to race-based disparities. The quantitative and qualitative data in this study will provide important context to generate hypotheses around the mechanisms for racial disparities in COVID-19, and may help to inform the development of multilevel strategies to reduce the burden of racial disparities in COVID-19 and its ongoing sequelae. Incorporating contextual information, elucidated from qualitative interviews, will increase the efficacy, adoption, and sustainability of such strategies.

### Comparison to Prior Work

Previous work examining racial disparities in COVID-19–related outcomes has largely been limited to quantitative approaches describing the relative risk of COVID-19 or COVID-19–related outcomes in one race or ethnic group relative to another. Few studies to date have employed a mixed methods approach to comprehensively explore the underlying mechanisms of racial disparities in COVID-19–related outcomes. One known study, using data from the “Health, Ethnicity and Pandemic Survey” (N=2506), a nationally representative survey conducted in October 2020, reported that Black respondents were 6 times more likely to report experiences of racism during COVID-19 [29]. The experience of racism was related to where people lived (eg, “red” vs “blue” states, and racially homogenous neighborhoods), as well as individual-level factors such as being male, low education, and lack of access to the internet [29]. This study highlighted the importance of examining the multilevel factors contributing to racism, but did not expand this research to examine mechanisms and associations with COVID-19–related outcomes, a focus of the current research.

### Strengths and Limitations

The key strength of this study is the use of a large integrated health care system (KPGA) with a rich EMR data infrastructure that includes individual, interpersonal, community, and structural factors, providing a unique opportunity to disentangle the key multilevel mechanisms underscoring racial disparities in COVID-19 for which few other data sets are equipped to address. Furthermore, KPGA is a longitudinal data set, and includes inpatient, outpatient, and general health encounters, leading to greater generalizability than most hospital-based COVID-19 studies performed to date. Our research team has extensive expertise using EMR data for research purposes [30-35], including validation studies [36], and is well-equipped to address the nuances of EMR data in research settings.

However, there are some limitations of this study to consider. First, KPGA has a higher proportion of Black adults compared to the Georgia population (41.6% and 32.6%, respectively), higher socioeconomic status (ie, median income and social vulnerability) [37], and does not include uninsured or Medicaid patients. Therefore, results from this study cannot be generalized to the broader Georgia population, but rather to those within an integrated health care system such as KPGA. Despite this, pervasive racial, ethnic, and socioeconomic disparities exist within the KPGA population. For example, Kaiser Permanente has previously reported racial and socioeconomic disparities with respect to health and well-being [38], gastric cancer [39], smoking cessation [40], and diabetes care [41], and preliminary evidence suggests that Black members are twice as likely to experience housing instability, indicating that a social gradient exists within this integrated health system. Understanding the underlying mechanisms contributing to racial disparities in COVID-19 in a population with comparatively uniform access to care is the focus of this work, for which the KPGA data infrastructure is well-suited.

Second, there are known limitations to the use of EMR data for research purposes, not least of which pertains to diagnosis bias: there is likely a race-based bias in terms of who is being screened, tested, and subsequently diagnosed with comorbidities. However, EMR data outperform claims and self-reported data. Moreover, the use of EMR data from a large population allows us to tease out underlying mechanisms of racial disparities in COVID-19 that would not be possible in a smaller, more select cohort population.

Third, our survey response rate was only 3%, similar to other email-based recruitment surveys. Consequently, our survey population is more likely to be white, female, and older as compared to the general KPGA population, thus limiting the external generalizability of our findings. However, the internal validity of our analyses examining the relative contribution of various SDOH factors and COVID-19–related disparities within this population is unlikely to be comprised by this selection bias, and thus the results will still be informative and generate important hypotheses for future work.

Finally, qualitative findings will be limited to a small number of COVID-19–related contexts due to the sample size. Here, we have prioritized understanding the context of health care navigation among Black and white KPGA members with COVID-19, as interventions to improve access, and thus reduce racial disparities, within an integrated health care system may be more readily addressed.

### Future Directions

In this pilot study, we hope to generate new knowledge regarding underlying mechanisms of race-based disparities in COVID-19 outcomes to inform the development of future multilevel interventions aimed at reducing inequalities within integrated care settings. Further, KPGA shares the same data infrastructure with 18 other health systems across the United States (in 13 states and serving >28.4 million patients). This will allow us to expand our work to a multisite study across the United States examining the impact of COVID-19 in communities of color in the southeast and nationally.

### Conclusion

In conclusion, this study will investigate race-based disparities in COVID-19 outcomes, and the contributing roles and mediating pathways of individual-level and social (eg, structural racism, neighborhood environment) factors among a racially and socioeconomically diverse population of people enrolled within an integrated health system. A rigorous examination of social contexts and racial disparities in COVID-19 outcomes will contribute to the identification of factors that can inform continuing efforts to address racial disparities in the United States in the context of COVID-19.

## Appendix

Multimedia Appendix 1 Quantitative survey questions.

Multimedia Appendix 2 Qualitative interview guide.

## Abbreviations

CAB: community advisory board
EMR: electronic medical record
ICD-10: International Classification of Diseases, 10th revision
ICU: intensive care unit
IRB: Institutional Review Board
KPGA: Kaiser Permanente Georgia
NIMHD: National Institute of Minority Health and Health Disparities
SDOH: social determinants of health

## References

[1] Azar KMJ, Shen Z, Romanelli RJ, Lockhart SH, Smits K, Robinson S, Brown S, Pressman AR (2020). Disparities in outcomes among COVID-19 patients in a large health care system In California. Health Aff.

[2] Gold JAW, Wong KK, Szablewski CM, Patel PR, Rossow J, da Silva J, Natarajan P, Morris SB, Fanfair RN, Rogers-Brown J, Bruce BB, Browning SD, Hernandez-Romieu AC, Furukawa NW, Kang M, Evans ME, Oosmanally N, Tobin-D'Angelo M, Drenzek C, Murphy DJ, Hollberg J, Blum JM, Jansen R, Wright DW, Sewell WM, Owens JD, Lefkove B, Brown FW, Burton DC, Uyeki TM, Bialek SR, Jackson BR (2020). Characteristics and clinical outcomes of adult patients hospitalized with COVID-19 - Georgia, March 2020. MMWR Morb Mortal Wkly Rep.

[3] Killerby ME, Link-Gelles R, Haight SC, Schrodt CA, England L, Gomes DJ, Shamout M, Pettrone K, O'Laughlin K, Kimball A, Blau EF, Burnett E, Ladva CN, Szablewski CM, Tobin-D'Angelo M, Oosmanally N, Drenzek C, Murphy DJ, Blum JM, Hollberg J, Lefkove B, Brown FW, Shimabukuro T, Midgley CM, Tate JE, CDC COVID-19 Response Clinical Team (2020). Characteristics associated with hospitalization among patients with COVID-19 - Metropolitan Atlanta, Georgia, March-April 2020. MMWR Morb Mortal Wkly Rep.

[4] Mahajan U, Larkins-Pettigrew M (2020). Racial demographics and COVID-19 confirmed cases and deaths: a correlational analysis of 2886 US counties. J Public Health.

[5] Price-Haywood EG, Burton J, Fort D, Seoane L (2020). Hospitalization and mortality among Black patients and white patients with Covid-19. N Engl J Med.

[6] COVID-19 United States cases by county. Johns Hopkins University and Medicine Coronavirus Research Center.

[7] Webb Hooper M, Nápoles AM, Pérez-Stable EJ (2020). COVID-19 and racial/ethnic disparities. JAMA.

[8] NIMHD Minority Health and Health Disparities Research Framework. National Institutes of Health National Institute on Minority Health and Health Disparities.

[9] Minkler M, Wallerstein N (2008). Community-based participatory research: from process to outcomes.

[10] Kelly-Pumarol IJ, Henderson PQ, Rushing JT, Andrews JE, Kost RG, Wagenknecht LE (2018). Delivery of the research participant perception survey through the patient portal. J Clin Transl Sci.

[11] Hennink M, Hutter I, Bailey A (2020). Qualitative research methods.

[12] Krieger N (2014). Discrimination and health inequities. Int J Health Serv.

[13] Krieger N, Waterman PD, Kosheleva A, Chen JT, Carney DR, Smith KW, Bennett GG, Williams DR, Freeman E, Russell B, Thornhill G, Mikolowsky K, Rifkin R, Samuel L (2011). PLoS One.

[14] Ioannidis JPA, Powe NR, Yancy C (2021). Recalibrating the use of race in medical research. JAMA.

[15] Lin SS, Kelsey JL (2000). Use of race and ethnicity in epidemiologic research: concepts, methodological issues, and suggestions for research. Epidemiol Rev.

[16] Elliott M, Fremont A, Morrison P, Pantoja P, Lurie N (2008). A new method for estimating race/ethnicity and associated disparities where administrative records lack self-reported race/ethnicity. Health Serv Res.

[17] Bursac Z, Gauss CH, Williams DK, Hosmer DW (2008). Purposeful selection of variables in logistic regression. Source Code Biol Med.

[18] Garg S, Kim L, Whitaker M, O'Halloran A, Cummings C, Holstein R, Prill M, Chai SJ, Kirley PD, Alden NB, Kawasaki B, Yousey-Hindes K, Niccolai L, Anderson EJ, Openo KP, Weigel A, Monroe ML, Ryan P, Henderson J, Kim S, Como-Sabetti K, Lynfield R, Sosin D, Torres S, Muse A, Bennett NM, Billing L, Sutton M, West N, Schaffner W, Talbot HK, Aquino C, George A, Budd A, Brammer L, Langley G, Hall AJ, Fry A (2020). Hospitalization rates and characteristics of patients hospitalized with laboratory-confirmed coronavirus disease 2019 - COVID-NET, 14 States, March 1-30, 2020. MMWR Morb Mortal Wkly Rep.

[19] Andridge RR, Little RJ (2009). The use of sample weights in hot deck imputation. J Off Stat.

[20] Isong I, Rao S, Bind M, Avendaño M, Kawachi I, Richmond T (2018). Racial and ethnic disparities in early childhood obesity. Pediatrics.

[21] Jackson J, VanderWeele T (2018). Decomposition analysis to identify intervention targets for reducing disparities. Epidemiology.

[22] (2021). Racial Data Dashboard. The COVID Tracking Project at The Atlantic.

[23] Liu X, Wang L (2019). Sample size planning for detecting mediation effects: a power analysis procedure considering uncertainty in effect size estimates. Multivariate Behav Res.

[24] (2020). COVID-19 OBSSR Research Tools. National Institute of Health Office of Behavioral and Social Science Research.

[25] Sanders EB, Stappers PJ (2008). Co-creation and the new landscapes of design. CoDesign.

[26] Lewis S (2015). Qualitative inquiry and research design: choosing among five approaches. Health Promotion Practice.

[27] Francis JJ, Johnston M, Robertson C, Glidewell L, Entwistle V, Eccles MP, Grimshaw JM (2010). What is an adequate sample size? Operationalising data saturation for theory-based interview studies. Psychol Health.

[28] Fetters MD, Molina-Azorin JF (2019). A checklist of mixed methods elements in a submission for advancing the methodology of mixed methods research. J Mixed Method Res.

[29] Su D, Alshehri K, Ern J, Chen B, Chen L, Chen Z, Han X, King KM, Li H, Li J, Li Y, Michaud T, Shi L, Ramos AK, Wen M, Zhang D (2022). Racism experience among American adults during COVID-19: a mixed-methods study. Health Equity.

[30] Harding J, Andes L, Rolka D, Imperatore G, Gregg EW, Li Y, Albright A (2020). National and state-level trends in nontraumatic lower-extremity amputation among U.S. medicare beneficiaries with diabetes, 2000-2017. Diabetes Care.

[31] Harding J, Benoit S, Gregg E, Pavkov M, Perreault L (2020). Trends in rates of infections requiring hospitalization among adults with versus without diabetes in the U.S., 2000-2015. Diabetes Care.

[32] Harding J, Shaw J, Peeters A, Cartensen B, Magliano D (2015). Cancer risk among people with type 1 and type 2 diabetes: disentangling true associations, detection bias, and reverse causation. Diabetes Care.

[33] Harding J, Shaw J, Peeters A, Guiver T, Davidson S, Magliano D (2014). Mortality trends among people with type 1 and type 2 diabetes in Australia: 1997-2010. Diabetes Care.

[34] Schrager JD, Patzer RE, Kim JJ, Pitts SR, Chokshi FH, Phillips JS, Zhang X (2019). Racial and ethnic differences in diagnostic imaging utilization during adult emergency department visits in the United States, 2005 to 2014. J Am Coll Radiol.

[35] Wiley Z, Ross-Driscoll K, Wang Z, Smothers L, Mehta A, Patzer R (2022). Racial and ethnic differences and clinical outcomes of patients with coronavirus disease 2019 (COVID-19) presenting to the emergency department. Clin Infect Dis.

[36] Loh V, Harding J, Koshkina V, Barr E, Shaw J, Magliano D (2014). The validity of self-reported cancer in an Australian population study. Aust N Z J Public Health.

[37] QuickFacts Georgia. U.S. Census Bureau.

[38] Stiefel MC, Gordon NP, Wilson-Anumudu FJ, Arsen EL (2019). Sociodemographic determinants of health and well-being among adults residing in the Combined Kaiser Permanente Regions. Perm J.

[39] Bautista MC, Jiang S, Armstrong MA, Kakar S, Postlethwaite D, Li D (2015). Significant racial disparities exist in noncardia gastric cancer outcomes among Kaiser Permanente's patient population. Dig Dis Sci.

[40] Young-Wolff KC, Adams SR, Tan AS, Adams AS, Klebaner D, Campbell CI, Satre DD, Salloum RG, Carter-Harris L, Prochaska JJ (2019). Disparities in knowledge and use of tobacco treatment among smokers in California following healthcare reform. Prev Med Rep.

[41] Marshall CJ, Rodriguez HP, Dyer W, Schmittdiel JA (2020). Racial and ethnic disparities in diabetes care quality among women of reproductive age in an integrated delivery system. Womens Health Issues.

