# Supplementary material for: Understanding Racial Disparities in COVID-19–Related Complications: Protocol for a Mixed Methods Study
Source: JMIR Res Protoc. 2022 Oct 6;11(10):e38914. doi: 10.2196/38914 (PMC9555818; doi:10.2196/38914)
Supplement: Multimedia Appendix 1 [file resprot_v11i10e38914_app1.pdf]

Appendix 1 – Survey

First, we would like to ask you some basic questions*. Choose the option that is most appropriate for you.

1. First Name
2. Last Name
3. What year were you born?
    - Option Year 1920-2003
4. What is your sex?
    - Male
    - Female
5. What is your Kaiser Permanente Georgia Medical Record Number (MRN)?
6. What race or races do you consider yourself to be? Select all that apply.
    - White
    - Black or African American
    - Asian
    - American Indian or Alaska Native
    - Native Hawaiian or Other Pacific Islander
    - Prefer not to answer
    - Do not know
7. Do you consider yourself to be Hispanic or Latino?
    - Yes
    - No
    - Prefer not to answer
    - Do not know
8. What is the highest degree or level of school you have completed?
    - Some high school
    - High school graduate or equivalent
    - Some college
    - Associate degree (for example AA, AS)
    - Bachelor's degree (for example, BA, BS, AB)
    - Graduate degree (for example, master's, doctorate, PhD)
9. What is your marital status?
    - Married
    - Divorced
    - Widowed
    - Separated
    - Never married
10. In 2019, what was your total household income before taxes?
    - Less than $25,000
    - $25,000-$34,999
    - $35,000-$49,999
    - $50,000-$74,999
    - $75,000-$99,999
    - $100,000-$149,999

- $150,000-$199,999
- $200,000 and above

**these variables are used for linkage purposes only.*

11. Since March 2020, have any of the following impacted your ability to receive healthcare (for any health concerns, including COVID-19)? Select all that apply.
    - You canceled an appointment
    - You postponed or delayed seeking care
    - You tried to get care but were turned away
    - Your provider canceled an appointment
    - Your provider postponed or delayed an appointment
12. Did you experience any symptoms of COVID-19?
    - Yes
    - No

[if answered yes to Q12]

12a. What symptoms did you experience? Select all that apply
    - Fever or chills
    - Cough
    - Shortness of breath
    - Chest pain
    - Sore throat
    - Headache
    - Muscle or body aches
    - Runny nose
    - Fatigue or excessive sleepiness
    - Confusion
    - Diarrhea
    - Nausea
    - Vomiting
    - Loss of sense of smell or taste

13. When you first thought you might have COVID-19, how long did it take you to get a COVID-19 test?
    - Less than 1 day (i.e., you took a test on the same day you thought you might have COVID-19)
    - 1-2 days
    - 3-4 days
    - 5-6 days
    - 7 or more days
14. Where did you go to get a COVID-19 test? (*KPGA = Kaiser Permanente Georgia)
    - KPGA clinic or testing site
    - Non-KPGA pharmacy (e.g. CVS or Wallgreens
    - Georgia Department of Health test site
    - Non-KPGA (privately own testing site (e.g. testing lab or non-KPGA clinic)
    - Hospital
    - Other

15. Where did you first go to seek medical care when you started experiencing symptoms of COVID-19 or thought that you might have COVID-19?
    - Registered medical practitioner in the community
    - Nearby public health facility or hospital
    - Nearby private clinic
    - Nearby private hospital
    - Traditional healer
    - Community health worker
    - Other
    - Did not seek medical care

Now, we are going to ask you some questions about your health BEFORE you were diagnosed with COVID-19.

16. Before you were diagnosed with COVID-19, how would you have rated your overall physical health?
    - Excellent
    - Very good
    - Good
    - Fair
    - Poor
17. Before you were diagnosed with COVID-19, how much sleep would you say you were getting, on average, every night?
    - Less than 6 hours
    - 6-8 hours
    - 8-10 hours
    - More than 10 hours
18. Before you were diagnosed with COVID-19, on average, how many days per week did you engage in moderate to strenuous exercise (e.g. a brisk walk)?
    - 1
    - 2
    - 3
    - 4
    - 5
    - 6
    - 7
19. Before you were diagnosed with COVID-19, on average, how many minutes per exercise session did you engage in moderate to strenuous exercise (e.g., a brisk walk)?
    - Option 1-150 minutes
20. Before you were diagnosed with COVID-19, did you smoke tobacco?
    - Yes - Frequently
    - Yes – Infrequently
    - No - But I have smoked in the past
    - No – Never
    - No – But I am exposed to tobacco via secondhand/passive/environmental smoke

[if answered yes to Q20]

20a. How many cigarettes a day do you smoke?

- 10 cigarettes or less
- 11-20
- 21-30
- 31 or more

21. Before you were diagnosed with COVID-19, how often, on average, did you drink alcohol?

- Never
- Monthly or less
- 2-4 times per month
- 2-3 times per week
- 4 or more times per week

[if answered yes to Q21]

21a. Before you were diagnosed with COVID-19, on average, how many alcoholic drinks did you have on a typical day when you were drinking?

- 1-2 drinks
- 3-4 drinks
- 5-6 drinks
- 7-9 drinks
- 10 or more drinks

21b. Before you were diagnosed with COVID-19, on average, how often did you have six or more alcoholic drinks on one occasion?

- Never
- Less than monthly
- Monthly
- Weekly
- Daily or almost daily

Now, we are going to ask you some questions about your health since being diagnosed with COVID-19. These questions relate to your health at the PRESENT TIME.

22. After you were diagnosed with COVID-19, how would you have rated your overall physical health?

- Excellent
- Very good
- Good
- Fair
- Poor

23. After you were diagnosed with COVID-19, how much sleep would you say you were getting, on average, every night?

- Less than 6 hours
- 6-8 hours
- 8-10 hours
- More than 10 hours

24. After you were diagnosed with COVID-19, on average, how many days per week did you engaged in moderate to strenuous exercise (e.g., a brisk walk)?

- 1

- 2
- 3
- 4
- 5
- 6
- 7

25. After you were diagnosed with COVID-19, on average, how many minutes per exercise session did you engage in moderate to strenuous exercise (e.g., a brisk walk)?
    - Option 1-150 minutes
26. After you were diagnosed with COVID-19, did you smoke tobacco?
    - Yes - Frequently
    - Yes - Infrequently
    - No - But I have smoked in the past
    - No – Never
    - No – But I am exposed to tobacco via secondhand/passive/environmental smoke

[if answered yes to Q26]

26a. How many packs, on average, do you smoke per day?

- 10 cigarettes or less
- 11-20
- 21-30
- 31 or more

27. After you were diagnosed with COVID-19, how often, on average, did you have a drink containing alcohol?
    - Never
    - Monthly or less
    - 2-4 times per month
    - 2-3 times per week
    - 4 or more times per week

[if answered yes to Q27]

27a. On average, how many drinks containing alcohol did you have on a typical day when you were drinking?

- 1-2 drinks
- 3-4 drinks
- 5-6 drinks
- 7-9 drinks
- 10 or more drinks

27b. After you were diagnosed with COVID-19, on average, how often did you have six or more alcoholic drinks on one occasion?

- Never
- Less than monthly
- Monthly
- Weekly
- Daily or almost daily

Now, we are going to ask you some questions about how the COVID-19 pandemic has affected your personal life more generally.

28. Since March 2020, have you experienced a shift to remote working?
- Yes
- No

[if answered yes to Q28]
28a. On a scale of 1-5, where 1=”not impacted at all” and 5=”majorly impacted”, how much has a shift to remote working impacted your personal daily life?
- 1 (not impacted at all)
- 2
- 3
- 4
- 5 (majorly impacted)

29. Since March 2020, have you experienced a relationship breakdown?
- Yes
- No

[if answered yes to Q29]
29a. On a scale of 1-5, where 1=”not impacted at all” and 5=”majorly impacted”, how much has this relationship breakdown impacted your personal daily life?
- 1 (not impacted at all)
- 2
- 3
- 4
- 5 (majorly impacted)

30. Since March 2020, have you experienced job loss?
- Yes
- No

[if answered yes to Q30]
30a. On a scale of 1-5, where 1=”not impacted at all” and 5=”majorly impacted”, how much has this job loss impacted your personal daily life?
- 1 (not impacted at all)
- 2
- 3
- 4
- 5 (majorly impacted)

31. Since March 2020, have you experienced a decrease in personal income?
- Yes
- No

[if answered yes to Q31]
31a. On a scale of 1-5, where 1=”not impacted at all” and 5=”majorly impacted”, how much has this decrease in personal income impacted your personal daily life?
- 1 (not impacted at all)
- 2
- 3
- 4
- 5 (majorly impacted)

32. Since March 2020, have you experienced financial hardship?

- Yes
- No

[if answered yes to Q32]

32a. On a scale of 1-5, where 1=”not impacted at all” and 5=”majorly impacted”, how much has this financial hardship impacted your personal daily life?

- 1 (not impacted at all)
- 2
- 3
- 4
- 5 (majorly impacted)

33. Since March 2020, has someone you know died from COVID-19?
    - Yes
    - No

[if answered yes to Q33]

33a. On a scale of 1-5, where 1=”not impacted at all” and 5=”majorly impacted”, how much has this death impacted your personal daily life?

- 1 (not impacted at all)
- 2
- 3
- 4
- 5 (majorly impacted)

34. Since March 2020, have you become newly responsible for providing care to a loved one?
    - Yes
    - No

[if answered yes to Q34]

34a. On a scale of 1-5, where 1=”not impacted at all” and 5=”majorly impacted”, how much has becoming newly responsible for providing care to a loved one impacted your personal daily life?

- 1 (not impacted at all)
- 2
- 3
- 4
- 5 (majorly impacted)

35. Since March 2020, have you become newly responsible for overseeing school or education for school-age children (e.g. homeschooling or facilitating virtual school)?
    - Yes
    - No

[if answered yes to Q35]

35a. On a scale of 1-5, where 1=”not impacted at all” and 5=”majorly impacted”, how much has becoming newly responsible for overseeing school or education for school-age children impacted your personal daily life?

- 1 (not impacted at all)
- 2
- 3
- 4
- 5 (majorly impacted)

36. Since March 2020, have you become newly responsible for childcare/daycare for children?

- Yes
- No

[if answered yes to Q36]

36a. On a scale of 1-5, where 1="not impacted at all" and 5="majorly impacted", how much has becoming newly responsible for childcare/daycare for children impacted your personal daily life?

- 1 (not impacted at all)
- 2
- 3
- 4
- 5 (majorly impacted)

Now we are going to ask you some questions about the COVID-19 vaccine.

37. Have you received the COVID-19 vaccine or do you plan on getting the COVID-19 vaccine when it becomes available to you?
    - Yes – I already have received the vaccine
    - Yes – I plan on getting the vaccine when it is available to me
    - No – I have not received the vaccine and do not plan on getting the vaccine
    - Unsure
38. Are you concerned about the safety of the COVID-19 vaccine?
    - Yes
    - No
    - Unsure
39. Are you concerned about how well the COVID-19 vaccine will work?
    - Yes
    - No
    - Unsure
40. What concerns do you have about the COVID-19 vaccine? Select all that apply.
    - Immediate side effects from receiving the vaccine
    - Long-term side effects
    - How well the vaccine will protect me from COVID-19
    - How long the vaccine will protect me from COVID-19

41. Would you encourage your friends or family to get the vaccine?
    - Yes
    - No
    - Unsure

Now, we are going to ask you some questions about your experiences with the health care system more generally. This includes your experiences before, during and after your diagnosis of COVID-19.

42. Have you ever felt unfairly treated in getting medical care?
    - Yes
    - No
43. Have you ever felt that you were denied medical care or provided inferior or poor medical care?

- Yes
- No

44. Have you ever had to wait a long period of time before getting medical care?
    - Yes
    - No
45. Have you ever had trouble getting medical care from a specialist such as a heart doctor?
    - Yes
    - No

46. For the next set of statements, please indicate if you strongly disagree, disagree, agree, or strongly agree with each statement (select only one option for each item):

	Strongly disagree	Disagree	Agree	Strongly agree
When I make plans, I am almost certain that I can make them work				
Getting people to do the right thing depends upon ability; luck has nothing to do with it				
What happens to me is my own doing				
Many of the unhappy things in people's lives are partly due to bad luck				
Getting a good job depends mainly on being in the right place at the right time				
Many times I feel that I have little influence over the things that happen to me				

47. For the next set of statements, please indicate if you strongly disagree, disagree, agree or strongly agree with each statement

	Strongly disagree	Disagree	Agree	Strongly Agree
You had better be cautious when dealing with healthcare organizations				
Patients have sometimes been deceived or misled by healthcare organizations				
When healthcare organizations make mistakes, they usually cover it up				
Healthcare organizations have sometimes done harmful experiments on patients without their knowledge				
Healthcare organizations don't always keep your information totally private				
Sometimes, I wonder if healthcare organizations really know what they are doing				
Mistakes are common in healthcare organizations				

I trust that health care organizations will tell me if a mistake is made about my treatment				
Health care organizations often want to know more about your business than they need to know				
The patient's medical needs come before other considerations at health care organizations				
Health care organizations are more concerned about making money than taking care of people				
Health care organizations put the patient's health first				
Patients should always follow the advice given to them at health care organizations				
I typically get a second opinion when I am told something about my health				
I trust that health care organizations check their staff's credentials to make sure they are hiring the best people				
They know what they are doing at health care organizations				
I trust that health care organizations keep up with the latest medical information				
