# Supplementary material for: Understanding Racial Disparities in COVID-19–Related Complications: Protocol for a Mixed Methods Study
Source: JMIR Res Protoc. 2022 Oct 6;11(10):e38914. doi: 10.2196/38914 (PMC9555818; doi:10.2196/38914)
Supplement: Multimedia Appendix 2 [file resprot_v11i10e38914_app2.pdf]

Appendix 2 - Interview Guide

Study Aim 2b: Understand factors that facilitate and impede health seeking behaviors at the interpersonal, family, community, and healthcare levels among members with COVID. These interviews will focus on how members navigated care and linked to care.

Part 1: COVID Status

1. How did you first learn that you were COVID positive?
2. Walk me through me through your daily experiences with COVID.
3. Tell me about a time you had a question(s) about COVID and got help with that question. (**informational support)**
    a. Who did you go to?
    b. What made you feel comfortable going to that source?
    c. Why did you trust this source?
    d. What is this person's connection to healthcare or the medical field, if any?
    e. Other people in network in healthcare field?
4. Tell me about a time you had a question but did not get an answer.
    a. What made it difficult?
    b. What could have made it easier?

Part 2: Care Navigation:

Now I want to talk with you about your experiences and interactions with providers and all health care employees. This can include any doctors, nurses, physician assistants, or anyone else involved in your care.

1. Start with your first interactions and walk me through the time when you felt you were recovered from COVID.
2. When did you first seek care for COVID?
    a. What lead you to seek care?
    b. Walk me through your symptoms with COVID?
    c. How did it take place? Phone? In-person? Video? Chat with a doctor?
3. How long was the time between you suspected you had COVID to the time you sought care?
4. I want you to think about the timing of you seeking care. Do you think it was too soon? Just right? Or later than you would have preferred? I want you to answer on a scale of 1 to 10.
    a. 1=too early
        i. *If closer to 1:* what are the reasons you felt it was too early?
    b. 5=just right
        i. *If closer to 5:* what makes you feel like you got care at the right time?
    c. 10=too late
        i. *If closer to 10:* what makes you feel like you went later than preferred?
5. What were your first interactions with a health professional about your care related to COVID? Please describe the interaction.
    a. How were you treated during this early interaction?
    b. How satisfied were you with this interaction?

    c. Did you feel like your needs were met?

6. We understand that there may have been multiple discussions with healthcare providers as you dealt with COVID. Please tell me about your story after this first encounter with a healthcare provider.
7. How did the health care professionals explain your treatment to you?
    a. *Probe:* How clear was the explanation?
    b. *Probe:* What was unclear in the explanation?
    c. *Probe:* What did you have trouble understanding about your diagnosis?
    d. *Probe:* What could have improved that experience for you?
8. What challenges did you have adhering to the treatment plan by providers?
    a. How realistic was this plan for you?
    b. How confident were you in this plan?
9. What challenges did you experience getting access to care?
    a. Transportation?
    b. Knowing how to access care and where to call?
    c. Knowing where to go?
10. How often did you have to serve as your own health advocate during your time having COVID?
11. Please think about your experiences having COVID and other experiences with health care or trying to get health care. Could you share an experience you have had with being treated differently because of the color of your skin?
    a. *Probe:* How did this make you feel?
    b. *Probe:* How did this change how your next health care encounter?

Part 3. Parallel needs and competing priorities

12. While you were handling your own healthcare, what else where you concerned about?
    a. Work?
    b. School
    c. Health of others?
13. Who else were you responsible for during this time?
14. How did you handle your responsibilities during this time?

Part 4. COVID lessons learned

<u>We want to learn more about your perspective on COVID-19.</u>

15. What, if anything, could have helped you in navigating COVID-19 your care? Or focus on your care?
16. How much control did you feel you had over your healthcare?
    a. What could have helped you have more control?
    b. What made you feel like you did not have control?
17. Were there any resources you needed to protect yourself but did not have?
18. If you had to summarize your experiences with COVID-19, what would you say?
19. What advice would you give to others about dealing with COVID-19?
